# The impact of RNA secondary structure on read start locations on the Illumina sequencing platform

**DOI:** 10.1371/journal.pone.0173023

**Published:** 2017-02-28

**Authors:** Adam Price, Jaishree Garhyan, Cynthia Gibas

**Affiliations:** 1 Department of Bioinformatics and Genomics, University of North Carolina at Charlotte, Charlotte, NC, United States of America; 2 Jawaharlal Nehru University, New Delhi, India; 3 Department of Immunity and Infectious Diseases, Forsyth Institute, Cambridge MA, United States of America; Texas A&M University College Station, UNITED STATES

## Abstract

High-throughput sequencing is subject to sequence dependent bias, which must be accounted for if researchers are to make precise measurements and draw accurate conclusions from their data. A widely studied source of bias in sequencing is the GC content bias, in which levels of GC content in a genomic region effect the number of reads produced during sequencing. Although some research has been performed on methods to correct for GC bias, there has been little effort to understand the underlying mechanism. The availability of sequencing protocols that target the specific location of structure in nucleic acid molecules enables us to investigate the underlying molecular origin of observed GC bias in sequencing. By applying a parallel analysis of RNA structure (PARS) protocol to bacterial genomes of varying GC content, we are able to observe the relationship between local RNA secondary structure and sequencing outcome, and to establish RNA secondary structure as the significant contributing factor to observed GC bias.

## Introduction

Single-stranded RNA molecules are known to fold into complex three dimensional structures that vary depending on the molecular sequence [1]. It has been shown that these structures are more than simply an artifact of free energy interactions occurring on an unstable single-stranded molecule, and that they are in some cases necessary for function [2, 3]. Many methods have been developed to predict the folded conformations of RNA molecules, and several computational methods have become popular in recent years, such as MFold [4] and Vienna RNA [5], which make predictions of RNA folding conformations based on free energy calculations. An experimental method for detecting secondary structure across the entire transcriptome, called PARS, has also been developed recently [6]. These technologies and methods make it possible to further investigate the role and effects of secondary structure.

Here, we investigate the effect RNA secondary structure has on gene expression data that is generated through modern sequencing technologies. It has been previously shown that there is a detectable dependence of read depth on GC content [7]. This effect has been observed in high throughput sequencing technologies, such as Illumina sequencers, that use PCR as a method of read replication [8]. It has been observed that regions of RNA with higher GC content have more stable secondary structures than RNA strands with lower GC content [9]. It has also been shown that the speed at which polymerase moves along an associated RNA strand is dependent on the secondary structure the polymerase encounters, and that polymerase works at a slower pace when confronted with more secondary structure elements [3]. Because the frequency of stable secondary structure increases as GC content increases, the relative abundance of high GC reads produced by next-gen sequencing is likely to be lower due to intermittent pausing by polymerase during fragment amplification. Additionally, amplification methods that rely on single stranded DNA with an associated primer correctly annealing to an oligo, such as flow cell cluster amplification in Illumina sequencing, may be subject to additional bias due to the initial single stranded fragments forming stable structures at the end of the strand.

In our study, we hypothesize that RNA secondary structure formation is the underlying cause of GC bias. We use the PARS assay to measure RNA secondary structure for three bacterial strains with varied levels of GC content (low, medium and high GC content). We show that secondary structure is the GC-correlated molecular property that impacts apparent gene expression levels as measured by transcript abundance. We identify the extent of this effect and use that information to statistically model the relationship between GC content, RNA secondary structure, and the abundance of reads produced by Illumina sequencers.

## Materials and methods

**Fig 1:**
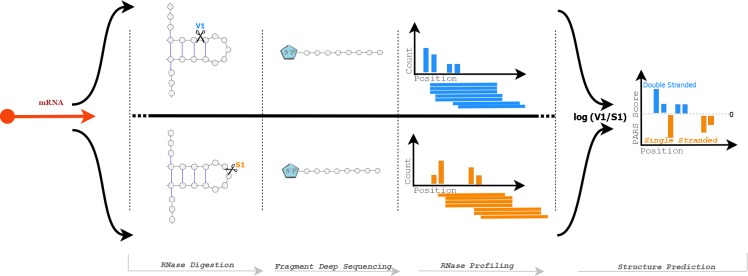
Selective enzyme digestion is performed on each organism using S1 RNase and V1 RNase. V1 RNase cuts selectively at double stranded positions, while S1 cuts at single stranded positions. A 5’ phosphate is attached to the fragment and deep sequencing is performed. The resulting data is then processed into profiles based on read start positions for each condition and then combined to create structural predictions.

The goal of this study was to examine the relationship between secondary structure in RNA transcripts and gene expression levels, with particular regard to GC content. To achieve this, it was first necessary to determine the presence or absence of secondary structure for the entire transcriptome at a nucleotide-level resolution. We employed the PARS method, a procedure for measuring transcriptome wide secondary structure [6] which has previously been demonstrated in Saccharomyces cerevisiae. PARS works by exposing RNA molecules to enzymes that selectively cleave them depending on their folded state. A sample of RNA was divided and one part was exposed to RNase V1 and another to RNase S1. RNase V1 randomly fragments double-stranded RNA, leaving behind a 5’ phosphate at the cleavage site. RNase S1 works similarly but targets single-stranded RNA. Adaptors that can ligate to these 5’ phosphoryl-terminated RNAs were then used to select against random fragmentation. The result is that for the RNase V1 sample, the starting position of the aligned read corresponds to a double-stranded region of RNA, and similarly RNase S1 read start sites corresponds to a single-stranded region. In order to convert this information into a quantitative measure at single nucleotide resolution, the log ratio of the number of reads starting at any given position is calculated based on the RNase V1 and RNase S1 read sets. A higher log ratio, also referred to as a PARS score, indicates a higher probability that nucleotides at a given position are in double-stranded conformations, while a lower score indicates a higher probability of single-stranded conformations [6]. An overview of this process can be seen in Fig 1.

Three gram positive bacterial strains with varying levels of genome-wide GC content were chosen from the from NCBI database for the PARS assay. *Staphylococcus epidermidis ATCC 12228*, with 32% GC, was chosen as a representative strain for low GC content, *Exiguobacterium sp*. *AT1b ATCC-BAA 1283* was chosen as a medium GC content strain with 48.5% GC, and *Micrococcus luteus ATCC-4698* was chosen as a representative strain for high GC with 73% GC content. These strains were selected because they had similar genomic sizes and fairly simple culture requirements.

### PARS assay

*S*. *epidermidis* was cultured in LB, while *M*. *luteus* and *Exiguobacterium sp*. *AT1b* were cultured in tripticase soy agar. All three cultures were grown to log phase and two volumes of RNA Protect were added to the culture. The cells were pelleted and the resulting pellet was either used directly for RNA extraction or saved at -80C for no longer than twelve hours following extraction. Cell lysis was carried out by freshly made ultrapure lysostaphin, or 125 μl lysostaphin + 200 μl TE NaCl + 5 μl proteinase K in 15 ml cell pellets. The cell pellet mix was vortexed with RLT and 0.1 mm for fifteen to thirty minutes using the disruptor genie vortexer. Similar pretreatment was done for *Exiguobacterium sp*. *AT1b*, except that ultrapure Lysozyme was used in place of Lysostaphin during the lysis phase. For *M*.*luteus* an incubation period of thirty minutes was optimized for lysis with lysozyme.

The RNAeasy midi kit (Qiagen) was used for final extraction of total RNA from the bacterial cell’s DNA contamination was then removed by treating the total RNA with 4–8 units of DNAse I twice at 37C for thirty minutes in a total volume of 50 μl, with a total nucleic acid concentration of 10 μg. Total RNA was checked for DNA contamination and integrity before proceeding with mRNA enrichment and PCR was subsequently carried out to check for DNA contamination after the DNAse treatment. Agilent 2100 bioanalyzer RNA 600 nano chip was used for total RNA integrity and quantification. Total RNA with RIN equal or greater than 9 was only further used for further experiments. mRNA enrichment was carried out using MICROBExpress kit (Ambion, Thermofisher Scientific) followed by QC on agilent bioanalyzer. mRNA was used to prepare directional paired end libraries of size 300 bp using modified methodology of directional library preparation and the TruSeq small RNA library preparation kit protocol (illumina Inc.). Briefly, after fragmentation by S1/V1 enzymes, the 5' P end was capture by ligating to 5' adapters and only those fragments which have 5'P end were captured in the cDNA and thus captured in the library. The final libraries were Ampure cleaned and ran on DNA 100 chip for QC before 200bp sequencing.

Three replicates of each sample and condition were prepared and sequenced using the Illumina HiSeq 2500 sequencer. In addition, a control condition was also performed for each organism, in which RNA-seq was performed using standard protocols. Three replicates were also used for the control condition.

### Data processing and selection

Raw sequence reads were aligned to their respective reference genomes using bowtie2 [10], with all samples showing a high rate of overall alignment. The resulting files generated by bowtie2 were then filtered for quality and converted to sorted BAM format using samtools [11]. This process was performed for each of the three conditions: S1 RNase digestion, V1 RNase digestion, and a control sample, for each of the three bacteria used in the experiment. This process was performed on all available replicate data sets, and replicate data were then merged. The data were subsequently used to create a file tallying the number of read start sites for each position along the transcriptome. These positional values were converted into PARS scores for each position along the transcriptome. The data were then filtered to select a subset of genes that had both the highest confidence PARS scores, due to having adequate data from each experimental condition, and few positions with unknown conformations. To select the most reliable data, data were filtered using a method based on the previously calculated positional values for all three organisms. For each position, in cases where positional data was absent in an experimental condition or in the control condition, a value of 0 was assigned. If data were present in both experimental conditions and in the control condition, PARS scores, the log ratio of the positional data for the two experimental conditions, were calculated for that position. Then, if the PARS score for the position had an absolute value of 3 or greater, a value of 1 was assigned to the position. Positions assigned 1 were considered to be high quality positions, and positions assigned 0 were considered to be poor quality positions. Finally, the percentage of high quality positions for each coding region was calculated, and regions with at least 80% high quality positions were retained for analysis. After this filtering step, fifty-nine genes were selected from *Staphylococcus epidermidis*, sixty-one genes were selected from *Exiguobacterium sp*., and eighty-one genes were selected from *Micrococcus luteus*.

## Results

### Secondary structure and read depth

In order to test one of the main hypotheses of this paper, that secondary structure is a possible cause of bias in the measurement of gene expression via high throughput sequencing, we first performed correlation testing. We compared read depth in a standard RNA-seq assay for each organism, to structural predictions at single nucleotide resolution generated in the PARS assay. The 23S rRNA genes for each organism were selected for initial inspection, as these genes were identified as having high quality data for all three organisms in the filtering step. We used Pearson’s product-moment correlation testing on these genes to examine the relationship between read depth in the control experiment, and PARS scores. In all three cases, significant correlation was found between PARS scores and read depth in the control samples that indicated higher levels of reads were present in positions that were predicted to be in secondary structure conformation. Correlation was found with Exiguobacterium sp. having a p-value of 8.944e-05, Staphylococcus epidermidis having a p-value of 0.04939, and Micrococcus luteus having a p-value less than 2.2e-16. This result supports the hypothesis that RNA secondary structure contributes to bias in read depth.

### Secondary structure and GC bias

**Fig 2:**
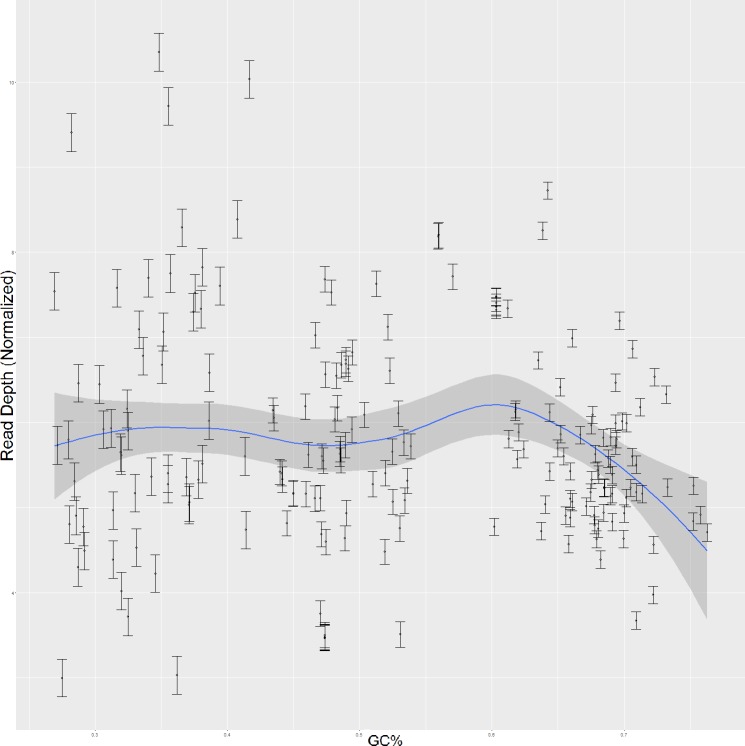
Normalized read depth in relation to GC content for 201 genes across all three organisms.

We next examined the relationship between GC bias and RNA secondary structure. It has been shown previously that Illumina sequencers exhibit a bias in which A-T residues are sequenced with higher frequency than are G-C residues [7]. It has been further shown that elevated GC content negatively influences sequencing coverage overall, with regions having GC content greater than 70% becoming increasingly read sparse [12]. As such, our investigation of GC content and secondary structure first attempted to confirm this finding, in order to validate subsequent findings in our data. Using the same subset of high-quality genes selected in the analysis of secondary structure’s effect on read depth, read depth as generated from the standard RNA-seq assays was contrasted with GC content. The results, shown in Fig 2, show regions of lower GC content having a more widely varied range of read depth, and regions of higher GC content showing a much more narrow range of read depth, with a tendency toward the lower end relative to the dataset as a whole. Pearson’s product-moment correlation test was performed on these data as a whole to characterize this relationship between GC content and read depth. The results of this test showed significant correlation between GC content and read depth with a p-value of 0.01436, which is consistent with previous research. A closer examination of the data shown in Fig 2 shows that regions with low GC content, less than 40%, is not correlated with read depth. However, as GC content increases, an increasingly strong relation between read depth and GC content can be identified. For regions with medium GC content, between 35% and 60%, a modest correlation between read depth and GC percentage is identified with a p-value of .02722. For regions of high GC content, however, the relationship is much stronger. Regions with greater than 60% GC content showed a strong inverse correlation with read depth with a p-value of 0.000004638. This result is consistent with previous research which has shown that regions with high GC content are more read sparse than medium and low GC content regions [12].

Next, data for each strain were individually analyzed using linear regression analysis. GC content in Staphylococcus epidermidis, the low GC strain, was not significantly correlated with read depth. However, Exiguobacterium sp., the medium GC strain, showed significant correlation between read depth and GC content with a p-value of 0.03323. Micrococcus luteus, the high GC strain, showed the strongest relationship between GC and read depth with a p-value of 0.0000130. Again, we observe that the relationship between GC content and read depth strengthens as the GC content of a strain increases. The discrepancy between the significance levels of these tests is also to be expected, as the magnitude of GC bias correspondingly varies with the composition of each organism. Staphylococcus epidermidis has an overall GC content of 32% genome-wide, which should not be strongly influenced by GC bias. The lack of a significant relationship between GC content and read depth in this organism shows that without the influence of GC bias that reads are more evenly distributed across a wider range of depth. Micrococcus luteus, having an average GC level of 48.5%, has a modest, though significant correlation between read depth and GC content. This level of correlation again shows the relationship between GC content and read depth, as some regions are beginning to be influenced by GC bias. In the case of this organism, the level of GC content is just high enough that GC bias becomes apparent, though low enough that the effect is not excessive. Exiguobacterium sp., with a 73% GC content level shows the strongest relation. Here, the effects of GC bias are apparent with a strongly significant correlation indicated. The reads are sparser and with increasing GC the read depth is correspondingly lower. The combination of these results confirms the presence of GC bias in our data and provides a basis for understanding the strength of the effect.

**Fig 3:**
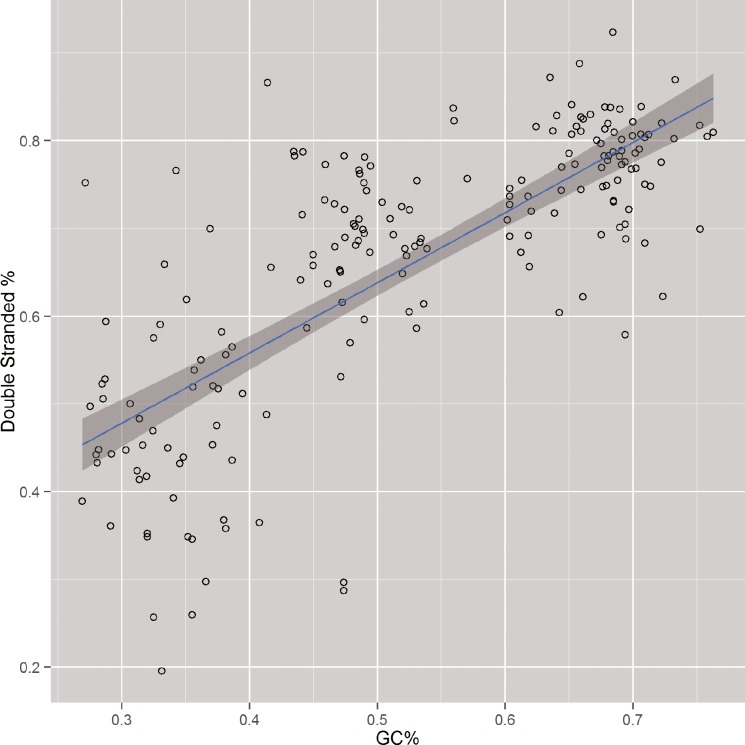
Percent of double-stranded predictions by PARS in relation to GC content for 201 genes across all three organisms.

Finally, correlation testing was performed to compare the percentage of positions predicted to be in a folded conformation with the percentage of GC content for each respective gene. The result of this analysis, shown in Fig 3, was highly significant, with a correlation of 74.1% and a p-value of less than 2.2e-16. This strong relationship between read depth and GC content, in combination with the previously shown capacity of read depth as a predictor of secondary structure conformation, indicate RNA secondary structure as a strong contributing factor to GC bias.

### Secondary structure and read start bias

Next, we investigated the hypothesis that bias is introduced due to secondary structure formation at fragment ends. To do this, binomial logistic regression was chosen to model the relationship between experimentally predicted secondary structure and the number of reads starting at corresponding positions in the control data. Binomial logistic regression is used to model dichotomous output variables as a function of predictor variables, and makes it possible to measure if a predictor variable affects an outcome variable and to what extent [13]. The outcome variable is this case is binary: a position is in a secondary structure conformation or a position is in a single stranded conformation. The predictor variable is the number of reads starting at the same position as measured by the control data. In this way, we are able to ask not only if there is a significant relationship between levels of reads produced and the presence of secondary structure, but to what extent it is expected that secondary structure exists at a position based on the number of reads starting at that position.

Secondary structure conformational predictions were first calculated using PARS scores representing folded states or single-stranded states for each position of each gene. In this step, positions identified by PARS scores as being in secondary structure conformations were assigned a value of 1, and positions predicted to be single stranded were assigned a value of 0. Logistic regression was then performed, wherein the logistic regression coefficients calculated represented a change in the log odds for a one unit increase in the predictor variable. In this case, for an increase of one in our predictor variable, the number of reads starting at this position in the control experiment, the log odds of that position being in a secondary structure conformation, increased by a factor equal to computed logistic regression coefficient. Using these logistic regression coefficients, confidence intervals and predicted probabilities were calculated across the range of possible values of for read starts. This made it possible to model the relationship between read starts and RNA secondary structure at a nucleotide resolution.

**Fig 4:**
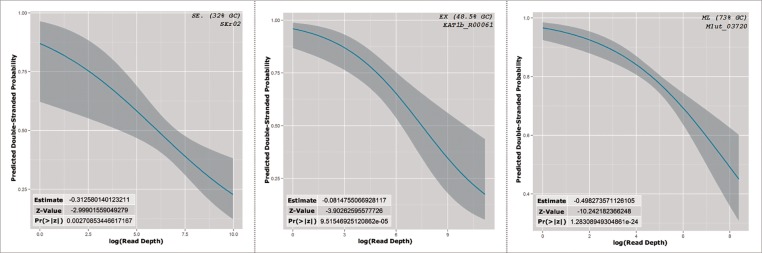
PARS predicted probabilities of secondary structure with regard to read starts in 23S rRNA genes for each organism.

Fig 4 shows these predictions for the 23S rRNA genes of each organism. This result showed a clear pattern for all three organisms, in which the predicted probability of a position being in secondary structure decreased as the number of reads starting at that position increased. This result indicates that positions that are known to fold into secondary structure conformations typically have fewer associated read starts.

### Summary of results

**Fig 5:**
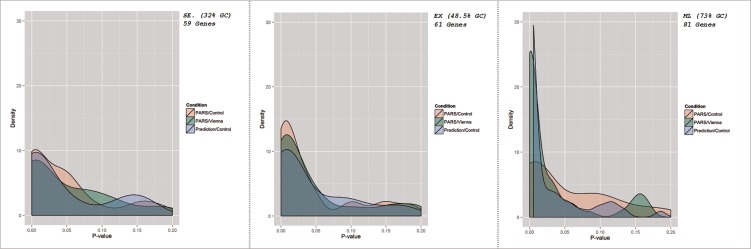
Distribution of correlation and regression testing results for all genes passing quality threshold. Read depth and experimentally predicted PARS score correlation is represented as PARS/Control (red), PARS/Vienna shows correlation results from computationally modelled secondary structure predictions and experimentally computed structures (green), and Prediction/Control shows correlation significance based on Logistic Regression (blue).

A summary of the analyses performed for each of the 207 genes that passed the quality criterion previously discussed and a summary of the results can be seen in Fig 5. The PARS/Control subheading (pink), shows the distribution of p-values for correlation testing between experimentally predicted secondary structure by PARS and read depth in the control experiment. The PARS/Vienna subheading (green) shows the results of correlation testing between experimentally measured secondary structure and free energy secondary structure predictions as calculated by the software package, Vienna2 [5]. The prediction/control subheading (blue) indicates the distribution of p-values from the binomial logistic regression analysis. In all cases a majority of the genes studied showed significance across all three tests, with the significance becoming increasingly apparent with higher levels of GC content.

## Discussion

GC content bias in has been shown to exist predominately in the stages before sequencing, specifically at the PCR stage [14]. However, the cause of GC bias exists as a combination of factors. It has been shown that polymerase pauses at the base of, or slightly before, secondary structure formation [15]. Furthermore, changes in RNA secondary structures that occur after the structure is only partially unwound lead to the formation of new secondary structural elements downstream from the first, which leads to slower progress overall by polymerase [16]. Other research has shown that sequences leading to stable secondary structure at fragment ends can cause adapter ligation to fail, leading to the failure of reverse transcription and introducing bias between the relative expression levels of fragments with secondary structure and those without [17]. The combination of these behaviors lead to a reduction in amplification efficiency, particularly in PCR based systems where relatively small differences before amplification may be represented exponentially afterwards.

Efforts to correct for GC bias involving methods of eliminating secondary structure formation have proven to be effective. Betaine is a zwitteronic osmoprotectant that alters DNA stability in such a way that stable secondary structure in GC rich regions melt at temperatures similar to those required to melt AT rich regions, and the introduction of betaine to PCR assays has been shown to suppress replication pausing by polymerase [18]. It has also been observed that increasing denaturing time in combination with the introduction of betaine substantially increases read coverage and depth in GC rich regions [14]. These findings, in conjunction with the findings of this study, indicate that the formation of RNA secondary structure is the primary cause of GC bias.

Although reduction in levels of secondary structure addresses a substantial portion of GC bias, it does not eliminate bias in sequencing assays completely. The use of betaine, for example, while reducing secondary structure in GC rich regions, may cause early disassociation of the newly synthesized strand from AT rich templates [14]. In one study, PCR-free FRT-seq was used, in which reverse transcription occurred directly on the flow cell after adapter ligation [19]. Comparison of optimized PCR assays with the PCR-free experiment showed that the optimized assay performed nearly as well as eliminating the PCR phase altogether [14]. Both assays, however, still produce fewer reads in GC rich regions, indicating that correction methods are as of yet imperfect or that other processes contribute to GC bias in ways that are not well understood.

In this paper we have generated a novel dataset that provides experimentally measured RNA secondary structure predictions at a nucleotide resolution and have used that data to examine the role of RNA secondary structure on GC bias as observed in modern sequencing technologies. We have demonstrated that our dataset is consistent with previous research findings, and that the relationship between GC content and read depth in three bacterial strains spans a wide range of GC content. We have described the relationships between RNA secondary structure and GC content and between RNA secondary structure and read depth. We have shown that fragment counts are significantly biased, with a lower frequency of read starts at sites which are folded into RNA secondary structure conformations. Finally, we have shown that the relationship between read depth and GC content causes increasing bias as GC content increases, with less significant biases being caused in lower GC sequences.

One possible limitation of this method is that V1 nuclease can also cleave at stacked nucleotides formed due to intramolecular interaction at positions that are not double stranded [20]. However, investigation into the possible bias of V1 in the PARS protocol has shown that there is a very small bias towards particular regions along the transcript. However, it has been confirmed that signals generated by RNase V1 are highly distinct from those generated by RNase S1 and global inspection across all transcripts for the PARS protocol revealed that approximately 7 percent of V1 and S1 peaks are shared. These shared peaks could be the result of experimental noise introduced by nonspecific enzymatic activity, but could also correspond to dynamic RNA regions or transcripts that fold into more than one stable conformation [6]. We therefore believe that this is an acceptable limitation of the PARS method and by extension of this study and several others [6, 21, 22].

The findings of our study point to optimization of assays with regard to RNA secondary structure as an ideal method of reducing GC bias. The identification and modeling of read fragment bias at positions predicted to be in secondary structure conformations is particularly novel and opens new avenues of research for the correction of GC bias. Future work in this area may include the computational modeling of the relationship between read start sites and RNA secondary structure, as well as the application of corrections to read counts based on adjustments for observed start site bias.
